# Prevalence and age of diagnosis of neurodevelopmental conditions among Asian populations in Aotearoa New Zealand

**DOI:** 10.1186/s11689-026-09695-z

**Published:** 2026-04-23

**Authors:** Noriko K. Panther, Nicholas Bowden, Joanna Chu, Stephanie D’Souza, Sujata Saha, Hiran Thabrew, Laurie K. McLay

**Affiliations:** 1 Child Well-Being Research Institute, University of Canterbury, Christchurch, New Zealand; 2 Department of Women’s and Children’s Health, University of Otago, Dunedin, New Zealand; 3 Faculty of Health, Te Kaupeka Oranga, University of Canterbury, Christchurch, New Zealand; 4 Department of Social and Community Health, Faculty of Medical and Health Sciences, University of Auckland, Auckland, New Zealand; 5 Faculty of Arts and Education, University of Auckland, Auckland, New Zealand; 6 Department of Psychological Medicine, Faculty of Medical and Health Sciences, University of Auckland, Auckland, New Zealand

## Abstract

**Purpose:**

Asian populations are among the fastest-growing ethnic groups in Aotearoa New Zealand (NZ), yet little is known about the prevalence of neurodevelopmental conditions (NDCs) within these communities. The study compared the prevalence and age of diagnosis of NDCs (Attention Deficit Hyperactivity Disorder [ADHD], autism, communication and language disabilities [CLDs], intellectual disability [ID], motor disabilities [MDs], and specific learning disabilities [SLDs]) between NZ-born Asian and non-Asian populations, and differences across Asian subgroups.

**Methods:**

A national cross-sectional analysis was conducted using linked administrative microdata from the Integrated Data Infrastructure, covering the 2021/22 estimated resident population aged 0–24 years (*N* = 1,334,247). Following adjustment for socioeconomic factors, standardized NDC rates were calculated for Asian and non-Asian populations and Asian subgroups (Indian, Chinese, Southeast Asian, and Other Asian).

**Results:**

Lower standardized rates of NDCs were identified among Asian (2.85%, 95% CI [2.77, 2.94]) compared to non-Asian (4.52%, 95% CI [4.49, 4.56]) participants. Most notably, rates of ADHD (1.1%, 95% CI [1.05, 1.16] vs. 2.94%, 95% CI [2.91, 2.97]) and ID (0.34%, 95% CI [0.31, 0.38] vs. 0.58%, 95% CI [0.57, 0.60]) were significantly lower among Asian participants. Among Asian sub-groups, rates of NDCs were lowest for Chinese children, with particularly low rates of Autism, MDs and SLDs.

**Conclusion:**

Findings highlight substantial differences in NDC rates between NZ-born Asian and non-Asian ethnicities, suggesting that socioeconomic context, cultural perceptions, and diagnostic pathways may influence identification patterns across and between Asian subgroups. Culturally responsive approaches are critical for equitable NDC identification and support.

## Introduction

Global awareness of neurodiversity is growing [37]. Neurodiversity refers to the naturally occurring neurological and neurocognitive variations within the human population [25]. The concept emphasizes inclusivity by recognizing that all individuals are neurodiverse, thereby valuing different ways of thinking, learning, and experiencing the world [25]. The term neurodivergence is used to describe the individual experience of these neurological differences. This encompasses a wide range of diagnoses, including attention-deficit/hyperactivity disorder (ADHD), autism, communication and language disabilities (CLDs), intellectual disability (ID), motor disabilities (MDs), and specific learning disabilities (SLDs), as well as acquired neurological differences such as traumatic brain injury [2, 55]. These conditions affect personal, social, academic, and occupational functioning [2]. Their impact varies across individuals, and co-occurrence of different forms of neurodivergence (e.g., ADHD and autism) is common [2].

Aotearoa New Zealand (NZ) has become a leader in promoting understanding and acceptance of neurodiversity [14, 31]. NZ positions itself as a “non-disabling society”, one in which people with and without disabilities can pursue their goals and aspirations, supported by a collective commitment across government, communities, and society to foster full inclusion [30]. However, few studies have investigated the prevalence of neurodevelopmental conditions (NDCs) in NZ, and research focusing specifically on Asian populations within NZ is even more limited [7]. This is despite their status as the third-largest ethnic group, and evidence that they are among the fastest-growing populations in NZ [50]. Those who identify as being of Asian ethnicity are projected to increase from 15% of the national population in 2018 to 26% by the early 2040 s [50]. The Asian population in NZ is culturally and linguistically diverse, encompassing a wide range of languages, histories, migration experiences, religions, and belief systems [50]. Among Asian ethnicities in NZ, Chinese and Indian communities are the largest, representing 5.2% and 5.1% of the national population, respectively [50]. This is followed by Filipino (2.2%), Korean (0.8%), Fijian Indian (0.5%), and Japanese ethnicities (0.4%), among others [50].

Beyond population size, the demographic profile of Asian communities in NZ also reveals important social differences. Chinese and East Asian communities are generally more socioeconomically advantaged than Indian and South Asian families, who are more likely to live in high-deprivation areas and experience greater financial stress [36]. These differences reflect varying migration histories, settlement contexts, and employment opportunities among Asian subgroups. These sociocultural differences may shape how health and disability are understood, influencing help-seeking patterns, diagnosis pathways, and access to NDC services across Asian subgroups.

Over the past decade, the global prevalence of NDCs has substantially increased around the world [24, 41, 57]. While there is some variation across conditions, recent global estimates suggest that up to 15% of children have a NDC [11, 12]. Increased rates have partly been attributed to improvements in identification procedures and revised diagnostic criteria [5, 6]. Higher prevalence estimates are typically reported in countries with greater social and economic development, reflecting differences in healthcare access, service capacity, public awareness, and societal acceptance [52].

Existing research on the prevalence of NDCs is geographically imbalanced, with most studies conducted in Western contexts, particularly the United States and Northern Europe [57]. However, there is a well-recognized need to include non-Western populations in health research, prompting increased attention to the prevalence of NDCs in Asia and other regions [32]. Reported prevalence rates in Asian countries tend to be lower than those in Western countries [42, 57], although considerable cross-national variability exists, ranging from 0.01% in South Korea to 3.2% in Japan [17, 40]. This variation likely reflects both methodological and socio-contextual differences, including cross-country variability in diagnostic practices, professional training, and community awareness and attitudes [52, 57].

Considerable variation has also been observed across studies conducted within Asian countries. For instance, prevalence estimates of autism in the Shanghai and Jilin regions of China ranged from 0.076 to 1.08%, respectively [20, 51]. These discrepancies may reflect methodological factors (e.g., sampling methods and identification procedures) as well as socio-geographical variation between urban and rural settings [52], although evidence regarding the impact of socio-geographical factors on prevalence remains inconclusive [18, 38].

Understanding prevalence estimates, including differences across communities, helps ensure that health services are appropriately designed and meet the needs of the communities. Such evidence also informs future policy planning and resource allocation, so that services and support are directed to areas of greatest need. The primary objectives of this study were to use population-level linked administrative data to compare the prevalence of NDCs, including ADHD, autism, CLDs, ID, MDs, and SLDs, between Asian and non-Asian populations born in NZ, and to assess differences in prevalence within Asian subgroups. A secondary aim was to compare the age at first NDC diagnosis between Asian and non-Asian populations.

## Methods

### Integrated data infrastructure

This national cross-sectional study utilized microdata from the Integrated Data Infrastructure (IDI), a whole-population administrative dataset managed by Stats NZ [49]. The IDI comprises anonymized, probabilistically linked administrative data from government and non-government agencies, the census, and questionnaire-based socioeconomic surveys at the individual, household, and broader levels [27]. The de-identified IDI dataset can only be accessed via a secure virtual connection in approved research facilities [49].

### Participant population

We constructed a national sample of children and young adults aged 0–24 years (henceforth, children) and born in NZ from the estimated resident population (ERP) for the 2021/22 fiscal year. We used an established IDI-based method for constructing the ERP, capturing all those who were alive and residing in NZ as at June 30, 2022 [13]. The 2021/22 fiscal year was selected as it represented the most recent year with complete and reliable health data for the identification of NDCs. The study focused on those born in NZ to ensure comprehensive and consistent health-service data coverage. Those born overseas were excluded because pre-migration health information was unavailable in the IDI, which would limit diagnostic completeness. Moreover, NZ’s immigration policy requires applicants to meet an acceptable standard of health, which restricts people with disabilities, or their family members with disabilities, from immigrating [19]. Born-in-NZ status was determined based on Department of Internal Affairs birth registration data.

### Neurodevelopmental conditions

Ascertainment of NDCs followed the case identification method outlined in Bowden et al. [4]. This method used diagnostic codes and inference medication dispensing recorded across four linked health datasets: hospital admissions (the National Minimum Dataset [NMDS]), specialist mental health services (Programme for the Integration of mental health data [PRIMHD]), disability support services (Socrates), and pharmaceutical dispensing data (pharmaceutical collection). Conditions of interest were ADHD, autism, CLDs, ID, and MDs and SLDs. Individuals were classified as having an NDC if they met criteria in any linked dataset at any point during the study period (see Table 3 in the Appendix for case identification details).

### Ethnicity

Ethnicity was classified using the NZ Standard Classification 2005V2.1.0 [47]. The population was stratified into Asian and non-Asian using Level 1 ethnicity groupings. Level 2 groupings were employed to separate the Asian group into Indian, Chinese, Southeast Asian, and Other Asian, where possible [46]. The total concept ethnicity approach was employed, meaning individuals could identify in more than one Level 2 group.

### Sociodemographic characteristics

Sociodemographic information was derived from the IDI Personal Details Table. Age, categorized as 0–4, 5–9, 10–14, 15–19, and 20–24 years, was determined as of 30 June 2022. Sex was classified as male or female. Area-level deprivation was measured using the NZ Index of Deprivation (NZDep) 2023 [3] and urban/rural profile of residence using the Geographic Classification for Health (GCH) rurality measure [54]. These area-based measures were both determined based on the individual’s meshblock, a neighbourhood-sized area of roughly 40–60 households, as at 30 June 2022, using IDI address notification data.

### Statistical analysis

The distribution of demographic characteristics and observed NDC rates was calculated for Level 1 ethnicity and Level 2 Asian ethnicity. Standardized rates of NDCs were generated by fitting logistic regression models with each NDC category as the dependent variable and ethnicity as the independent variable, controlling for age, sex, deprivation, and urban/rural residence. The Stata margins command was used to calculate predicted probabilities of NDC for each individual for each ethnicity while holding other covariates at observed values. Averaging these probabilities produced standardized rates, interpreted as the prevalence of NDC in each ethnic group if they shared the same population composition as the national sample. This standardization approach adjusts for differences in age structure between groups but does not explicitly model person-time at risk or apply time-to-event methods. Differences in age at NDC identification between Asian and non-Asian groups were examined by plotting cumulative proportions of individuals identified with an NDC by age (in years) at first indication.

### Procedure

All analyses were undertaken in the secure Stats NZ IDI DataLab, following the “Five Safes” framework [48]. The study was approved by the University of Otago Human Research Ethics Committee (reference: HD17/004) and was reviewed as a “Minimal Risk Health Research—Audit and Audit related studies” proposal. Stats NZ approved access to the IDI (reference: MAA2017–16). Data management was undertaken in SAS EG 8.3 and analysis in Stata/MP 19.5.

## Results

The 2021/22 ERP of individuals aged 0–24 years contained 1,566,306 people. Among these, 232,059 (14.8%) were not born in NZ and thus excluded from the study; of those excluded, 97,026 (41.8%) were identified as Asian. This yielded an analytical sample of 1,334,247 individuals. Among the analytical sample, 173,070 (13.0%) were of Asian ethnicity. This included 64,002 (37.0%) Indian, 59,601 (34.4%) Chinese, 29,670 (17.1%) Southeast Asian, and 8,040 (4.6%) Other Asian (Table 1).

**Table 1:** Demographic characteristics of individuals Aged 0–24 years born in NZ, by ethnicity (2021/22 estimated resident population)

	Non-Asian		Asian		Indian		Chinese		SE Asian		Other Asian	
	n	%	n	%	n	%	n	%	n	%	n	%
Total	1,161,177		173,070		64,002		59,601		29,670		8,040	
Sex												
Female	566,343	48.8	84,804	49.0	31,317	48.9	29,151	48.9	14,556	49.1	3,930	48.9
Male	594,834	51.2	88,266	51.0	32,685	51.1	30,450	51.1	15,114	50.9	4,110	51.1
Age (Years)												
0–4	228,516	19.7	57,288	33.1	24,828	38.8	15,123	25.4	10,842	36.5	3,231	40.2
5–9	229,053	19.7	45,150	26.1	15,630	24.4	17,025	28.6	7,611	25.7	2,127	26.5
10–14	251,796	21.7	32,313	18.7	10,914	17.1	12,297	20.6	5,289	17.8	1,407	17.5
15–19	231,132	19.9	22,890	13.2	7,833	12.2	8,658	14.5	3,447	11.6	849	10.6
20–24	220,683	19.0	15,429	8.9	4,797	7.5	6,498	10.9	2,478	8.4	429	5.3
Deprivation Quintiles (Area level)												
1	205,389	17.7	33,000	19.1	10,038	15.7	14,871	25.0	4,461	15.0	1,107	13.8
2	202,980	17.5	35,892	20.7	11,259	17.6	15,342	25.7	5,226	17.6	1,212	15.1
3	211,542	18.2	35,976	20.8	12,429	19.4	13,083	22.0	6,060	20.4	1,590	19.8
4	230,049	19.8	35,622	20.6	14,829	23.2	9,468	15.9	7,083	23.9	1,947	24.2
5	308,232	26.5	32,187	18.6	15,309	23.9	6,681	11.2	6,786	22.9	2,166	26.9
Urban/Rural												
Urban	914,949	78.8	163,428	94.4	60,789	95.0	57,303	96.1	26,835	90.4	7,566	94.1
Rural	243,369	21.0	9,267	5.4	3,075	4.8	2,148	3.6	2,784	9.4	456	5.7

*SE Asian* Southeast Asian

Marked demographic differences were observed between Asian and non-Asian populations, particularly in age, deprivation, and urban/rural distributions (Table 1). Asian children were younger, with a high proportion in the 0–9 age range (59.2%), compared with non-Asian children (39.4%). In contrast, non-Asian populations had comparatively higher proportions in the older age groups, particularly those aged 15–24 years (38.9%) compared with Asian populations (22.1%). Deprivation patterns diverged considerably, with non-Asian children more likely to reside in the most deprived quintile (26.5%) than Asian children (18.6%). Substantial differences in urbanicity of residence were also observed, with a higher proportion of Asian children living in urban areas (94.4%) than non-Asians (78.8%).

Marked heterogeneity was also evident across Asian subgroups (Table 1). Most notably, Indian children were more likely than Chinese children to reside in the most deprived quintile (23.9% compared to 11.2%). Southeast Asian children had the highest proportion of children living in rural areas (9.4%). Other Asian children were more likely to be from the youngest age groups.

There was a clear difference between Asian and non-Asian populations in the observed prevalence of NDCs (Table 2). Overall, Asian populations showed lower rates of NDCs (2.4%) than non-Asians (4.6%). This gap was particularly pronounced for ADHD, where prevalence among non-Asians (3.0%) was more than three times higher than among Asians (0.9%). ID was also more common among non-Asians (0.6%), compared with Asians (0.2%). In contrast, the prevalence of autism, CLDs, MDs, and SLDs was observed at similar levels between Asian and non-Asian groups (1.3% vs 1.3% for autism, 0.3% vs 0.2% for CLDs, and 0.8% vs 0.8% for MDs and SLDs). Observed overall NDC prevalence rates were broadly consistent across Asian subgroups, ranging from 2.3% among Chinese and Indian populations to 2.6% among Southeast Asian and Other Asian groups. Observed rates of specific NDCs across Asian sub-groups were generally similar. The most notable exception was autism, where Southeast Asians had a higher observed rate (1.6%), compared to the other Asian sub-groups.

**Table 2:** Observed NDCs rates by ethnicity

Ethnicity	Any NDC		ADHD		Autism		CLDs		ID		MDs & SLDs	
	n	%	n	%	n	%	n	%	n	%	n	%
Non-Asian	53,799	4.6	35,055	3.0	15,501	1.3	2,730	0.2	7,098	0.6	9,636	0.8
Asian	4,119	2.4	1,551	0.9	2,193	1.3	462	0.3	402	0.2	1,422	0.8
Indian	1,470	2.3	528	0.8	762	1.2	183	0.3	177	0.3	582	0.9
Chinese	1,386	2.3	591	1.0	690	1.2	132	0.2	114	0.2	423	0.7
SE Asian	783	2.6	252	0.8	468	1.6	81	0.3	69	0.2	261	0.9
Other Asian	213	2.6	72	0.9	114	1.4	33	0.4	24	0.3	69	0.9

*NDC* Neurodevelopmental condition, *ADHD* Attention-deficit/hyperactivity disorder, *CLDs* Communication and language disabilities, *ID* Intellectual disability, *MDs* Motor disabilities, *SLDs* Specific learning disabilities, *SE Asian* Southeast Asian

**Fig. 1:**
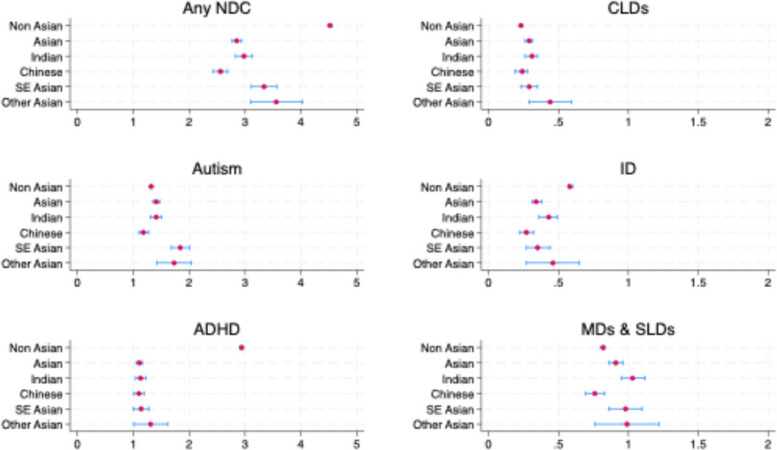
NDC rates by ethnicity standardized by age, sex, area-level deprivation and urban/rural**.** *Note*. NDC = Neurodevelopmental condition; ADHD = Attention-deficit/hyperactivity disorder; CLDs = communication and language disabilities; ID = intellectual disability; MDs = motor disabilities; SLDs = specific learning disabilities; SE Asian = Southeast Asian

After adjusting for socioeconomic factors, the Asian population demonstrated a significantly lower standardized prevalence of any NDCs (2.85%, 95% CI: 2.77–2.94) than the non-Asian population (4.52%, 95% CI: 4.49–4.56) (Fig. 1; see also Table 4 in the Appendix). The standardized prevalence rates for specific NDCs between Asian and non-Asian populations largely followed patterns consistent with observed rates. This included significantly higher rates of ADHD and ID among the non-Asian population. After adjustment, however, the rate of autism was significantly higher among the Asian population, compared to the non-Asian population, albeit by a small magnitude (1.41%, 95% CI: 1.35–1.47 vs 1.32%, 95% CI: 1.30–1.34). Likewise, rates of CLDs, MDs, and SLDs were also slightly higher among the Asian population. Within the Asian population, the overall rate of any NDCs was significantly lower among Chinese. Similarly, Chinese also had lower rates of autism, MDs, and SLDs. ADHD was highest among Other Asians.

**Fig. 2:**
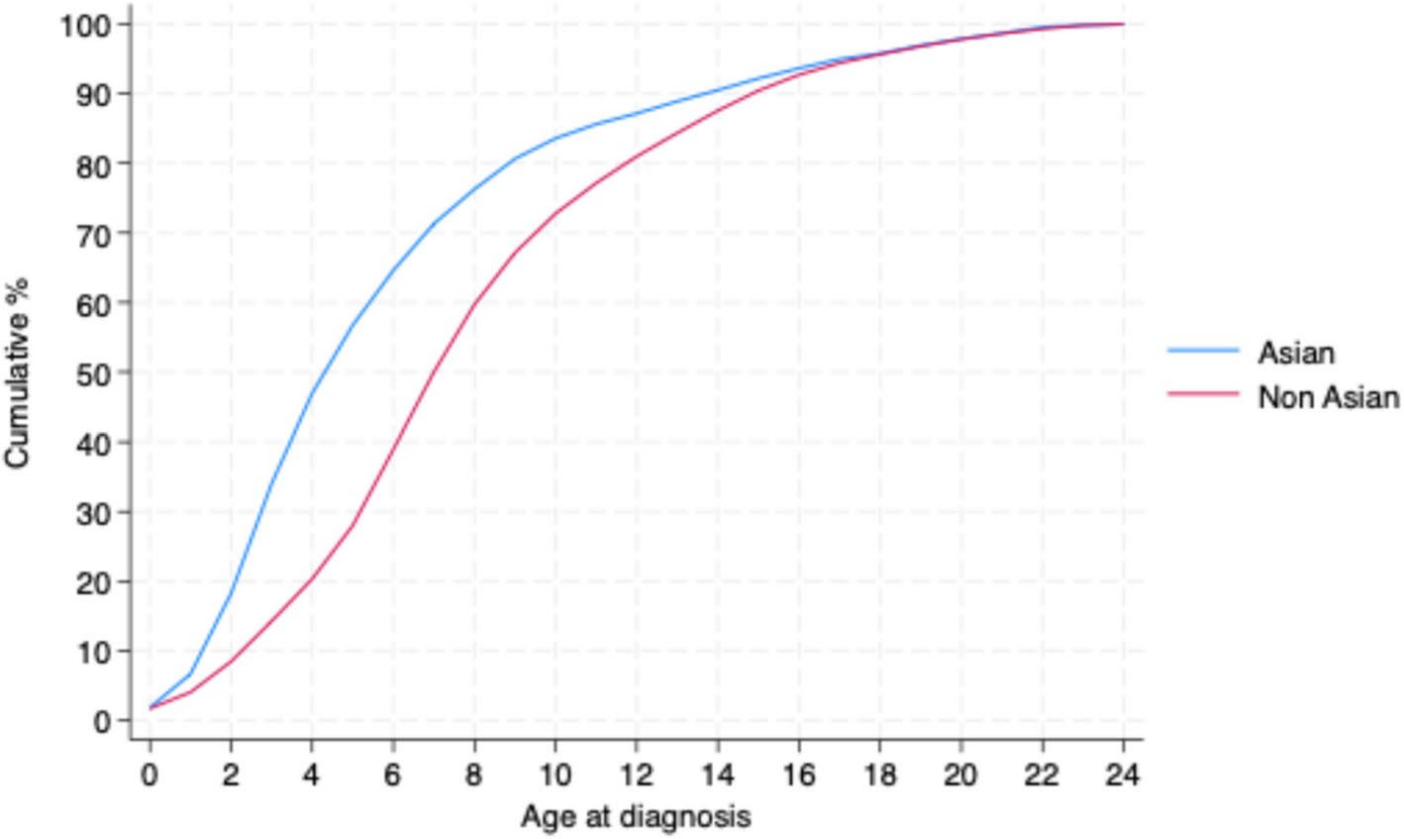
Age at diagnosis of any NDC of Asian and non-Asian ethnicities

There was a clear disparity between Asian and non-Asian populations in the age at diagnosis of any NDCs (Fig. 2; see also Table 5 in the Appendix). Asian populations were diagnosed earlier than non-Asians with a median age of five years at diagnosis compared to seven years. Over half (56.7%) of the Asian population with NDCs were identified before six years of age, compared with 28.0% among non-Asian populations. Among Asian children, 26.9% were diagnosed in middle childhood (ages 6–10 years) and 8.6% in adolescence (ages 11–15 years). By contrast, non-Asian populations had substantially higher proportions diagnosed in middle childhood (45.0%) and adolescence (17.7%).

## Discussion

This whole-of-population study compared the prevalence and age of diagnosis of NDCs among NZ-born Asian and non-Asian populations in NZ. Using linked administrative data, among the 2021/22 ERP of individuals aged 0–24 years, we identified marked differences across groups. Following adjustment for socioeconomic factors, lower overall rates of NDCs (2.8%) were identified among Asian than non-Asian participants (4.5%). However, interesting differences were noticed between these groups regarding NDC subtypes, with rates of most NDCs including autism (1.4 vs 1.3), CLDs (0.3 vs 0.2), and MDs and SLDs (0.9 vs 0.8) in fact being slightly higher among Asian participants, while rates of ADHD (1.1 vs 2.9) and ID (0.3 vs 0.6) were significantly lower compared to non-Asian participants. Chinese had lower overall NDCs rates compared to other Asian subgroups including lower rates of autism, ID, MDs and SLDs. Among those identified with an NDC, the age at diagnosis was younger for Asian compared to non-Asian participants.

These findings highlight significant disparities in the prevalence of NDCs among Asian and non-Asian children. The substantially lower NDC rate among Asian children was primarily driven by lower recorded rates of ADHD and aligns with international trends [42, 57]. It may not only reflect systemic factors such as diagnostic pathways and identification practices, but also cultural differences in help-seeking behaviors, beliefs about parenting, and attitudes towards NDCs, all of which can contribute to delayed or missed identification [10, 44, 57].

Despite the overall difference between groups being skewed by ADHD results, Asian children were in fact slightly more likely to be diagnosed with autism (1.4 vs 1.3), CLDs (0.3 vs 0.2), and MDs and SLDs (0.9 vs 0.8) than their non-Asian counterparts. This contrasts with earlier IDI-based autism estimates, which reported a population rate of 0.57% but a lower rate among Asian children (0.45%) using data to 2015/16. The substantially higher rate observed in the present study (1.3%) likely reflects improved case ascertainment with extended data coverage to 2021/22, the exclusion of individuals born overseas, and broader increases in autism identification over time. It was also notable that over half of the Asian families in this study received an NDC diagnosis by age five, compared with 28% of non-Asian children. Earlier diagnoses of NDCs among Asian children may reflect the presentation of more pronounced or clearly observable characteristics—cases that are less likely to be obscured by bilingual or multilingual language development [22]. These findings may also reflect that Asian parents are more likely to recognize social-communication differences characteristic of autism and other NDCs than ADHD as requiring professional support, since such differences are often more noticeable, creating more social distance [35]. Families with higher socioeconomic status (SES) may be particularly sensitive to these social-communication difficulties in cultural contexts that place a high value on school readiness and academic achievement [23]. By contrast, ADHD-related behaviors, including hyperactivity and impulsivity, may be perceived as reflections of parenting practices, particularly approaches to discipline, rather than as indicators of an underlying neurodevelopmental difference [34]. ADHD-associated inattention may also be obscured when co-occurring with other NDCs such as autism. The combination of these factors may mean that ADHD is underdiagnosed among Asian children, as opposed to being less prevalent in this group.

Several socioeconomic and cultural factors also likely contribute to the lower observed prevalence of NDCs among Asian compared to non-Asian populations. Most Asian people (74.9%) are born overseas, and nearly half (48.9%) are recent migrants who arrived within the past decade (Stats NZ, 2023). Recent migrants often face cultural and linguistic barriers that limit access to health and educational services, potentially leading to under-identification of NDCs [21]. Sociocultural and systemic factors may also play a role in rates of diagnosis across NDCs. The diagnostic pathways for autism often involve specialist assessments initiated through early childhood services, while ADHD diagnoses in NZ typically arise through primary care or school referrals, which may be less accessible to migrant families [31, 45]. Together, these factors could contribute to the differing diagnostic profiles observed across ethnic groups.

Chinese families showed a lower overall standardized rate of any NDCs, including autism, ID, and MDs and SLDs, compared with Indian, Southeast Asian, and Other Asian groups. Chinese families were the most socioeconomically advantaged, with over half living in the least deprived areas (50.7%) and only a quarter (27.1%) in the most deprived. By comparison, nearly half of Indian (47.1%) and Southeast Asian (46.8%) families and over half of Other Asians (51.0%) lived in the most deprived areas. This pattern is consistent with international findings, which typically associate lower SES with higher NDC rates [8].

This disparity between Chinese and other Asian subgroups likely reflects a complex interplay of socioeconomic and cultural factors. While all children in this study were born in NZ, their parents were likely migrants, given that a large proportion of Asian residents in the NZ population are overseas-born (Stats NZ, 2023). Chinese migrants tend to arrive through skilled migration pathways and are often highly educated, while frequently experiencing challenges navigating Western medical and educational systems due to cultural and linguistic differences [21, 29]. Across studies, Chinese New Zealanders consistently report lower healthcare utilization and greater cultural barriers than other Asian subgroups, despite higher SES [1, 36, 39]. In contrast, Indian and Southeast Asian groups show higher engagement with health and educational services but face more structural and economic barriers [33, 36]. Taken together, these patterns may contribute to under-identification of NDCs among Chinese families, while higher detection among Indian and Southeast Asian families may occur despite the presence of structural barriers.

These differences in prevalence may also reflect broader cultural attitudes towards NDCs. Chinese cultures are often characterised as collectivist, emphasising mutual responsibility and maintaining social harmony [53]. Within this context, having a family member with a disability may be perceived as challenging the family’s identity and social standing [16]. Consequently, Chinese families with higher SES may experience greater stigma surrounding NDCs within their communities and may prefer private or non-clinical support pathways. They may also be more reluctant to disclose conditions such as autism, ID, and MDs and SLDs, which tend to be more socially visible than ADHD, potentially contributing to under-diagnosis [35]. The Other Asian group includes smaller East Asian subpopulations (e.g., Japanese and Korean) that share similar cultural values with Chinese communities, though their small numbers and heterogeneity may obscure these patterns. 

While higher engagement with health and educational services among Indian and Southeast Asian families may have contributed to greater identification of NDCs, this does not necessarily indicate fully inclusive community environments. Stigma toward NDCs has also been documented in these communities, where parents of children with NDCs frequently encounter social, emotional, and financial challenges, including prejudice, discrimination, and social exclusion [9, 26]. However, given the limited cross-cultural evidence on attitudes toward NDCs, further research is needed to understand how cultural frameworks shape diagnostic practices across different Asian communities.

Collectively, these findings underscore the importance of culturally responsive approaches to identifying and supporting Asian children with NDCs and their families in NZ. Clinicians should be aware that presentations of ADHD may differ cross-culturally, with the inattentive subtype being more common in Asian cultures and less likely to attract teacher or parent concern [10]. Likewise, it is important for referrers to be aware of cultural differences in perceptions of NDCs, understanding of child development, and help-seeking patterns. Further, early developmental screening and family education should highlight the full spectrum of characteristics of NDCs, while accounting for cultural differences in language, attention, and communication patterns. For Asian families, especially recent migrants, clinical pathways must incorporate culturally appropriate engagement strategies, such as multilingual assessment, family-centered communication, and collaboration with community organizations [56]. Tailored outreach, together with culturally informed clinician and educator training and collaboration, are crucial in reducing diagnostic delays, promoting equitable access to interventions and educational support, and fostering trust between Asian families and service providers.

This study offers important insights while also highlighting several areas for further methodological development. First, ascertainment of NDCs relied solely on administrative health data available within the IDI. A major constraint is the absence of primary care records and limited information on the initial diagnosis of conditions. This is likely to have led to undercounting of NDCs overall, though the extent of under-ascertainment is unknown and may vary across condition types. In addition, case identification relies on ICD-10-AM and DSM-IV diagnostic codes, as DSM-5 classifications are not available within the IDI. These older diagnostic frameworks, including codes that do not map directly onto current conceptualisations of autism, may introduce some heterogeneity or misclassification within the identified cohort. Furthermore, as the case-identification method has not been formally validated, the balance of false negatives and false positives cannot be determined. Second, for ADHD specifically, we used dispensing of stimulant medication as a proxy for diagnosis. While this approach is well established in the literature [5, 6, 15, 43], it is more prone to misclassification. Third, there are limitations related to ethnicity data. Although we aimed to reflect the diversity of the Asian population in Aotearoa, small numbers meant results could only be presented for Indian, Chinese, Southeast Asian, and Other Asian groups. This necessarily falls short of capturing the full cultural and linguistic diversity within the Asian population, and the findings should therefore be interpreted with caution. Finally, while models adjusted for age and standardised estimates to a common age distribution, we did not explicitly account for differences in person-time at risk (i.e., time available to receive a diagnosis) across individuals. As such, younger individuals in the cohort have had less opportunity for diagnosis than older individuals, and this may influence observed prevalence estimates. Despite these limitations, IDI-based administrative sources provide a valuable foundation for generating evidence about Asian populations in Aotearoa NZ, contributing to filling existing evidence gaps and respond to current demands for cultural equity. In addition, administrative data offer important advantages, including the ability to analyze small or hard-to-reach populations and to investigate relatively rare outcomes with greater statistical power [28].

## Conclusions

This study found marked ethnic differences in the prevalence and age of diagnosis of NDCs among Asian and non-Asian populations in NZ. Asian children had lower overall rates of NDCs, seemingly driven by lower rates of ADHD. However, differences existed between NDC subtypes, with rates of autism, CLDs, MDs and SLDs slightly higher among Asian participants. Chinese participants showed lower overall rates of NDCs, including lower rates of autism, ID, and MDs and SLDs relative to other Asian subgroups. These differences likely reflect sociocultural and systemic factors influencing recognition and access to assessment. Culturally and linguistically responsive approaches to referral, screening and diagnosis are essential to reduce under-identification, improve equity in early detection, and ensure that Asian families receive appropriate and timely developmental support.

## Data Availability

All data supporting the findings of this study are available within the paper and its Supplementary Information.

## Appendix

**Table 3:** ICD-10-AM, DSM-IV, SOCRATES, and pharmaceutical codes used to identify neurodevelopmental condition (NDC) groups

NDC group	Code (Description)
	ICD-10-AM Codes (NMDS and PRIMHD)
ADHD	F900 (disturbance of activity and attention)
ADHD	F908 (other kinetic disorders)
ADHD	F909 (hyperkinetic disorder, unspecified)
Autism	F840 (childhood autism)
Autism	F841 (atypical autism)
Autism	F843 (other childhood disintegrative disorder)
Autism	F845 (Asperger’s syndrome)
Autism	F848 (other pervasive developmental disorders)
Autism	F849 (pervasive developmental disorder, unspecified)
CLD	F801 (expressive language disorder)
CLD	F802 (receptive language disorder)
CLD	F808 (other developmental disorders of speech and language)
CLD	F809 (developmental disorder of speech and language, unspecified)
CLD	F800 (specific speech articulation disorder)
FASD	P043 (foetus and new-born affected by maternal use of alcohol)
FASD	Q860 (foetal alcohol syndrome (dysmorphic))
ID	F700 (mild intellectual disabilities)
ID	F701 (mild intellectual disabilities)
ID	F708 (mild intellectual disabilities)
ID	F709 (mild intellectual disabilities)
ID	F710 (moderate intellectual disabilities)
ID	F711 (moderate intellectual disabilities)
ID	F719 (moderate intellectual disabilities)
SLMD	F810 (specific reading disorder)
SLMD	F811 (specific spelling disorder)
SLMD	F812 (specific disorder of arithmetical skills)
SLMD	F813 (mixed disorder of scholastic skills)
SLMD	F818 (other developmental disorders of scholastic skills)
SLMD	F819 (developmental disorder of scholastic skills, unspecified)
SLMD	F82 (specific development disorder of motor function) (dyspraxia)
SLMD	R480 (dyslexia and other symbolic dysfunctions)
SLMD	R481 (agnosia)
SLMD	R482 (apraxia)
SLMD	R488 (other and unspecified symbolic dysfunctions)
SLMD	R278 (other and unspecified lack of coordination)
	DSM-IV codes (PRIMHD)
ADHD	31400 (attention deficit hyperactivity disorder, predominantly inattentive type)
ADHD	31401 (attention deficit hyperactivity disorder, combined type)
ADHD	3149 (attention deficit hyperactivity disorder, NOS)
Autism	29900 (autistic disorder)
Autism	29910 (childhood disintegrative disorders)
Autism	29980 (pervasive developmental disorder – NOS)
CLD	31531 (expressive language disorder)
CLD	3070 (stuttering)
CLD	31539 (phonological disorder)
CLD	3079 (communication disorder NOS)
ID	317 (mild mental retardation)
ID	3180 (moderate mental retardation)
ID	3181 (severe mental retardation)
ID	3182 (profound mental retardation)
ID	319 (unspecified mental retardation)
SLMD	315 (reading disorder)
SLMD	3151 (mathematics disorder)
SLMD	3152 (disorder of written expression)
SLMD	3154 (developmental coordination disorder)
SLMD	3159 (learning disorder NOS)
	Diagnosis codes (SOCRATES)
ADHD	1201 (attention deficit/hyperactivity, e.g. ADD, ADHD)
Autism	1211 (autistic spectrum disorder (ASD))
Autism	1206 (Asperger’s syndrome)
Autism	1207 (retired - other autistic spectrum disorder (ASD))
CLD	1701 (mute)
CLD	1202 (language delay)
CLD	1799 (other speech disorder, specified)
CLD	1203 (speech delay)
CLD	9006 (social communication disorder)
FASD	1106 (foetal alcohol syndrome)
ID	1208 (intellectual disability, type not specified)
SLMD	1205 (dyslexia)
SLMD	1209 (learning disability, type not specified)
SLMD	1210 (developmental delay, type not specified)
SLMD	1299 (other intellectual, learning or developmental disorder)
SLMD	1204 (motor delay, developmental dyspraxia)
	Chemical codes (Pharmaceutical collection)
ADHD	1389 (Dexamfetamine sulfate)
ADHD	1809 (Methylphenidate hydrochloride)
ADHD	3880 (Methylphenidate hydrochloride extended release)
ADHD	3998 (Atomoxetine)

**Table 4:** NDC rates by ethnicity among those born in NZ, standardized by age, sex, deprivation and urban/rural

Ethnicity	Any NDC	ADHD	Autism	CLDs	ID	MDs & SLDs
Non-Asian	4.52 (4.49, 4.56)	2.94 (2.91, 2.97)	1.32 (1.30, 1.34)	0.23 (0.22, 0.24)	0.58 (0.57, 0.60)	0.82 (0.80, 0.83)
Asian	2.85 (2.77, 2.94)	1.11 (1.05, 1.16)	1.41 (1.35, 1.47)	0.29 (0.26, 0.31)	0.34 (0.31, 0.38)	0.91 (0.86, 0.96)
Indian	2.98 (2.83,3.13)	1.13 (1.04,1.23)	1.41 (1.31,1.51)	0.31 (0.26,0.35)	0.43 (0.36,0.49)	1.03 (0.95,1.12)
Chinese	2.56 (2.43,2.69)	1.10 (1.01,1.19)	1.18 (1.10,1.27)	0.24 (0.19,0.28)	0.27 (0.22,0.32)	0.76 (0.69,0.83)
SE Asian	3.34 (3.11,3.57)	1.14 (1.00,1.28)	1.84 (1.68,2.01)	0.29 (0.23,0.35)	0.35 (0.27,0.44)	0.98 (0.86,1.10)
Other Asian	3.56 (3.10,4.03)	1.31 (1.01,1.61)	1.73 (1.42,2.04)	0.44 (0.29,0.59)	0.46 (0.27,0.65)	0.99 (0.76,1.22)

*NDC* Neurodevelopmental condition, *ADHD* Attention-deficit/hyperactivity disorder, *CLDs* Communication and language disabilities, *ID* Intellectual disability, *MDs* Motor disabilities, *SLDs* Specific learning disabilities, *SE Asian* Southeast Asian

**Table 5:** Age at diagnosis of any NDC of Asian and non-Asian populations born in NZ

Age at Dx.	Asian		Non-Asian	
	n	%	n	%
0	75	1.82	915	1.70
1	198	4.82	1,254	2.33
2	477	11.61	2,373	4.41
3	648	15.77	3,117	5.79
4	531	12.92	3,270	6.08
5	402	9.78	4,134	7.68
6	324	7.88	5,874	10.92
7	276	6.72	5,964	11.08
8	204	4.96	5,217	9.70
9	180	4.38	4,014	7.46
10	120	2.92	3,024	5.62
11	84	2.04	2,355	4.38
12	63	1.53	2,067	3.84
13	72	1.75	1,806	3.36
14	66	1.61	1,731	3.22
15	69	1.68	1,551	2.88
16	60	1.46	1,218	2.26
17	54	1.31	909	1.69
18	33	0.80	672	1.25
19	48	1.17	621	1.15
20	39	0.95	543	1.01
21	33	0.80	438	0.81
22	36	0.88	366	0.68
23	12	0.29	270	0.50
24	6	0.15	102	0.19

*Dx* Diagnosis

## Abbreviations

ADHD: Attention deficit hyperactivity disorder
CLD: Communication and language disability
ERP: Estimated resident population
GCH: Geographic Classification for Health
ID: Intellectual disability
IDI: Integrated Data Infrastructure
MD: Motor disability
NDC: Neurodevelopmental condition
NMDS: The National Minimum Dataset
NZ: New Zealand
NZDep: New Zealand Index of Deprivation
PRIMHD: Programme for the Integration of Mental Health Data
SES: Socioeconomic status
SLD: Specific learning disability

## References

[1] Ameratunga S, Tin ST, Rasanathan K, Robinson E, Watson P. Use of health care by young Asian New Zealanders: Findings from a national youth health survey. J Paediatr Child Health. 2008;44(11):636–41. 18717771 10.1111/j.1440-1754.2008.01372.x

[2] American Psychiatric Association. Diagnostic and statistical manual of mental disorders: DSM-5 (5th ed.). American Psychiatric Association. 2013.

[3] Atkinson J, Salmond C, Crampton P, Viggers H, Lacey K. NZDep2023 Index of Socioeconomic Deprivation. In: University of Otago Wellington, New Zealand. 2024.

[4] Bowden N, Schluter PJ, Asaka U, Dacombe J, Hii J, Lee J, et al. Mortality risk of youth with neurodevelopmental conditions: an Aotearoa New Zealand nationwide birth cohort study. JAMA pediatrics. 2025;180(1):35–44. 10.1001/jamapediatrics.2025.4335 PMC12584066 41182792

[5] Bowden N, Thabrew H, Kokaua J, Audas R, Milne B, Smiler K, et al. Autism spectrum disorder/Takiwātanga: an integrated data infrastructure-based approach to autism spectrum disorder research in New Zealand. Autism. 2020;24(8):2213–27. 32677449 10.1177/1362361320939329 PMC7542998

[6] Bowden N, Thabrew H, Kokaua J, Braund R. National prescribing rates and polypharmacy for children and young people in New Zealand with and without autism spectrum disorder. Res Autism Spectr Disord. 2020;78:101642. 10.1016/j.rasd.2020.101642.

[7] Chiang A, Simon-Kumar R, Peiris-John R. A decade of Asian and ethnic minority health research in New Zealand: findings from a scoping review. N Z Med J. 2021;134(1542):67–83. 34531585

[8] de Araujo CM, Barbosa MG, Ramos AC, Santana VO, Silva I, Nordahl-Hansen A, et al. Relationship between structural brain differences and social factors associated with neurodevelopmental disorders: a systematic review. Neurosci Biobehav Rev. 2025;176:106266. 40540826 10.1016/j.neubiorev.2025.106266

[9] Desai MU, Divan G, Wertz FJ, Patel V. The discovery of autism: Indian parents’ experiences of caring for their child with an autism spectrum disorder. Transcult Psychiatry. 2012;49(3–4):613–37. 10.1177/1363461512447139. 22722980 10.1177/1363461512447139 PMC3472559

[10] Feng A, O’Neill S, Rostain AL. Contributors to underdiagnosis of ADHD among Asian Americans: a narrative review. J Atten Disord. 2024;28(12):1499–519. 39082427 10.1177/10870547241264113 PMC11912696

[11] Francés L, Quintero J, Fernández A, Ruiz A, Caules J, Fillon G, Hervás A, Soler CV. Current state of knowledge on the prevalence of neurodevelopmental disorders in childhood according to the DSM-5: a systematic review in accordance with the PRISMA criteria. Child Adolesc Psychiatry and Ment Health. 2022;16(1):27. 10.1186/s13034-022-00462-1. 10.1186/s13034-022-00462-1 PMC8973738 35361232

[12] Gidziela A, Ahmadzadeh YI, Michelini G, Allegrini AG, Agnew-Blais J, Lau LY, Duret M, Procopio F, Daly E, Ronald A. A meta-analysis of genetic effects associated with neurodevelopmental disorders and co-occurring conditions. Nat Hum Behav. 2023;7(4):642–56. 10.1038/s41562-023-01530-y PMC10129867 36806400

[13] Gibb S, Bycroft C, Matheson-Dunning N. Identifying the New Zealand resident population in the integrated data infrastructure (IDI). Statistics New Zealand= Tatauranga Aotearoa. 2016.

[14] Gray L, McNeill B, Pecora L, Macfarlane S, Hayley A, Hitch D, et al. Navigating neurodivergence: a scoping review to guide health professions educators. Med Educ. 2025;59(10):1037–48. 40130326 10.1111/medu.15676 PMC12438025

[15] Hobbs M, Bowden N, Marek L, Wiki J, Kokaua J, Theodore R, et al. The environment a young person grows up in is associated with their mental health: a nationwide geospatial study using the integrated data infrastructure, New Zealand. Soc Sci Med. 2023;326:115893. 37119566 10.1016/j.socscimed.2023.115893

[16] Holroyd EE. Chinese cultural influences on parental caregiving obligations toward children with disabilities. Qual Health Res. 2003;13(1):4–19. 12564260 10.1177/1049732302239408

[17] Hong M, Lee SM, Park S, Yoon S-J, Kim Y-E, Oh I-H. Prevalence and economic burden of autism spectrum disorder in South Korea using national health insurance data from 2008 to 2015. J Autism Dev Disord. 2020;50(1):333–9. 31630294 10.1007/s10803-019-04255-y

[18] Hsu Y-H, Chen C-W, Lin Y-J, Li C-Y. Urban–rural disparity in the incidence of diagnosed autism spectrum disorder in Taiwan: A 10-year national birth cohort follow-up study. J Autism Dev Disord. 2023;53(5):2127–37. 35132529 10.1007/s10803-022-05453-x

[19] Immigration New Zealand. Acceptable standard of health. 2025. https://www.immigration.govt.nz/about-us/news-centre/acceptable-standard-of-health/?utm_source=chatgpt.com.

[20] Jin Z, Yang Y, Liu S, Huang H, Jin X. Prevalence of DSM-5 autism spectrum disorder among school-based children aged 3–12 years in Shanghai, China. J Autism Dev Disord. 2018;48(7):2434–43. 29453711 10.1007/s10803-018-3507-z

[21] Kanengoni-Nyatara B, Watson K, Galindo C, Charania NA, Mpofu C, Holroyd E. Barriers to and recommendations for equitable access to healthcare for migrants and refugees in Aotearoa, New Zealand: an integrative review. J Immigr Minor Health. 2024;26(1):164–80. 37665540 10.1007/s10903-023-01528-8 PMC10771599

[22] Kay-Raining Bird E, Genesee F, Verhoeven L. Bilingualism in children with developmental disorders: a narrative review. J Commun Disord. 2016;63:1–14. 10.1016/j.jcomdis.2016.07.003. 27461977 10.1016/j.jcomdis.2016.07.003

[23] Kinlaw CR, Kurtz-Costes B, Goldman-Fraser J. Mothers’ achievement beliefs and behaviors and their children’s school readiness: a cultural comparison. J Appl Dev Psychol. 2001;22(5):493–506.

[24] Lai D-C, Tseng Y-C, Hou Y-M, Guo H-R. Gender and geographic differences in the prevalence of intellectual disability in children: analysis of data from the national disability registry of Taiwan. Res Dev Disabil. 2012;33(6):2301–7. 10.1016/j.ridd.2012.07.001. 22877930 10.1016/j.ridd.2012.07.001

[25] Legault M, Bourdon J-N, Poirier P. From neurodiversity to neurodivergence: the role of epistemic and cognitive marginalization. Synthese. 2021;199(5):12843–68.

[26] Liu W, Gong X, Ou J, Chen S. Burden and inequality of autism spectrum disorders in global, East Asian, and Southeast Asian regions, 1990–2021: result from the global burden of disease study 2021. BMC Public Health. 2025;25(1):2810. 40819035 10.1186/s12889-025-23904-9 PMC12357384

[27] Milne BJ, Atkinson J, Blakely T, Day H, Douwes J, Gibb S, et al. Data resource profile: the New Zealand integrated data infrastructure (IDI). Int J Epidemiol. 2019;48(3):677–677e. 30793742 10.1093/ije/dyz014

[28] Milne BJ, D’Souza S, Andersen SH, Richmond-Rakerd LS. Use of population-level administrative data in developmental science. Annu Rev Dev Psychol. 2022;4:447–68. 37284522 10.1146/annurev-devpsych-120920-023709 PMC10241456

[29] Ministry for Ethnic Communities. Ethnic evidence: Increasing the visibility and value of New Zealand's diversity. 2024. https://www.ethniccommunities.govt.nz/__data/assets/pdf_file/0023/63545/mecethnicevidencereport2024.pdf?utm_source=chatgpt.com.

[30] Ministry of Disabled People. New Zealand disability strategy 2016–2026. 2016. https://www.whaikaha.govt.nz/assets/About-us/Disability-Strategy/pdf-nz-disability-strategy-2016.pdf.

[31] Ministry of Disabled People. Aotearoa New Zealand autism guideline: He waka huia takiwātanga rau. 2022. https://whaikaha.govt.nz/about-us/policy-strategies-and-action-plans/NZ-autism-guideline/.

[32] Nair R, Chen M, Dutt AS, Hagopian L, Singh A, Du M. Significant regional inequalities in the prevalence of intellectual disability and trends from 1990 to 2019: a systematic analysis of GBD 2019. Epidemiol Psychiatr Sci. 2022;31:e91. 36539341 10.1017/S2045796022000701 PMC9805697

[33] New Zealand Immigration. New Zealand migrant settlement and integration strategy: Outcome indicators 2019. 2021. https://www.immigration.govt.nz/assets/inz/documents/employer-resources/2019-Settlement-Strategy-Supplementary-report.pdf?utm_source=chatgpt.com.

[34] Norvilitis JM, Fang P. Perceptions of ADHD in China and the United States: a preliminary study. J Atten Disord. 2005;9(2):413–24. 10.1177/1087054705281123. 16371664 10.1177/1087054705281123

[35] Park S, Lee Y, Kim CE. Korean adults’ beliefs about and social distance toward attention-deficit hyperactivity disorder, Tourette syndrome, and autism spectrum disorder. Psychiatr Res. 2018;269:633–9. 10.1016/j.psychres.2018.08.023 30212793

[36] Peiris-John R, Bavin L, Kang K, Dizon L, Lewycka S, Ameratunga S, et al. Factors predicting forgone healthcare among Asian adolescents in New Zealand: unmasking variations in aggregate data. N Z Med J. 2022;135(1549):63–80. 35728141

[37] Pellicano E, den Houting J. Annual research review: shifting from ‘normal science’ to neurodiversity in autism science. J Child Psychol Psychiatry. 2022;63(4):381–96. 10.1111/jcpp.13534. 34730840 10.1111/jcpp.13534 PMC9298391

[38] Raina SK, Chander V, Bhardwaj AK, Kumar D, Sharma S, Kashyap V, et al. Prevalence of autism spectrum disorder among rural, urban, and tribal children (1-10 years of age). J Neurosci Rural Pract. 2017;8(3):368–74. 10.4103/jnrp.jnrp_329_16. 28694615 10.4103/jnrp.jnrp_329_16 PMC5488556

[39] Rasanathan K, Ameratunga S, Chen J, Robinson E. A health profile of young Asian New Zealanders who attend secondary school. Adolesc Health. 2006;116:U380.

[40] Saito M, Sakamoto Y, Terui A. Epidemiology of ASD in Preschool-age Children in Japan. In Autism spectrum disorders-Recent advances and new perspectives. IntechOpen. 2022.

[41] Salari N, Ghasemi H, Abdoli N, Rahmani A, Shiri MH, Hashemian AH, et al. The global prevalence of ADHD in children and adolescents: a systematic review and meta-analysis. Ital J Pediatr. 2023;49(1):48. 37081447 10.1186/s13052-023-01456-1 PMC10120242

[42] Salari N, Rasoulpoor S, Rasoulpoor S, Shohaimi S, Jafarpour S, Abdoli N, et al. The global prevalence of autism spectrum disorder: a comprehensive systematic review and meta-analysis. Ital J Pediatr. 2022;48(1):112. 10.1186/s13052-022-01310-w. 35804408 10.1186/s13052-022-01310-w PMC9270782

[43] Schluter PJ, Bowden N, Dacombe J, McLay L, Lee M. Hospital dental admissions and caries experience among children with neurodevelopmental disabilities: a population-based record linkage cohort study. Community Dent Oral Epidemiol. 2025;53(2):160–9. 10.1111/cdoe.13018. 39533151 10.1111/cdoe.13018

[44] Shi Y, Guevara LRH, Dykhoff HJ, Sangaralingham LR, Phelan S, Zaccariello MJ, et al. Racial disparities in diagnosis of attention-deficit/hyperactivity disorder in a US national birth cohort. JAMA Netw Open. 2021;4(3):e210321–e210321. 33646315 10.1001/jamanetworkopen.2021.0321 PMC7921900

[45] Skirrow P, Cookson S, Bull D. Working together for change: ADHD diagnosis and treatment in New Zealand. J New Zealand College Clin Psychol. 2023;33(1):10–7.

[46] Stats NZ. Ethnicity New Zealand Standard Classification 2005V2.1.0. 2005. https://aria.stats.govt.nz/aria/#ClassificationView:uri=http://stats.govt.nz/cms/ClassificationVersion/YVqOcFHSlguKkT17.

[47] Stats NZ. Ethnicity New Zealand Standard Classification 2005 V2.1.0. 2020. https://aria.stats.govt.nz/aria/#ClassificationView:uri=http://stats.govt.nz/cms/ClassificationVersion/YVqOcFHSlguKkT17.

[48] Stats NZ. How we keep integrated data safe. 2022. https://www.stats.govt.nz/integrated-data/how-we-keep-integrated-data-safe/.

[49] Stats NZ Integrated Data Infrastructure (IDI). 2022 https://www.stats.govt.nz/integrated-data/integrated-data-infrastructure/.

[50] Stats NZ. 2023 census of population and dwellings. 2023. https://www.stats.govt.nz/2023-census/.

[51] Sun X, Allison C, Wei L, Matthews FE, Auyeung B, Wu YY, et al. Autism prevalence in China is comparable to Western prevalence. Mol Autism. 2019;10(1):7. 30858963 10.1186/s13229-018-0246-0 PMC6394100

[52] Talantseva OI, Romanova RS, Shurdova EM, Dolgorukova TA, Sologub PS, Titova OS, et al. The global prevalence of autism spectrum disorder: a three-level meta-analysis. Front Psychiatry. 2023;14:1071181. 36846240 10.3389/fpsyt.2023.1071181 PMC9947250

[53] Triandis HC. Collectivism and individualism as cultural syndromes. Cross-Cult Res. 1993;27(3–4):155–80.

[54] Whitehead J, Davie G, de Graaf B, Crengle S, Fearnley D, Smith M, Lawrenson R, Nixon G. Defining rural in Aotearoa New Zealand: A novel geographic classification for health purposes. 2021. 10.26635/6965.5495 35999779

[55] WHO. International Classification of Diseases: 11th revision (ICD-11). World Health Organization (WHO). 2025. https://icd.who.int/en/.

[56] Yang M, Parackal S, Gurung G, Subramaniam RM. Asian migrants navigating New Zealand primary care: a qualitative study. J Prim Health Care. 2023;15(1):30–7. 37000548 10.1071/HC22132

[57] Zeidan J, Fombonne E, Scorah J, Ibrahim A, Durkin MS, Saxena S, et al. Global prevalence of autism: a systematic review update. Autism Res. 2022;15(5):778–90. 35238171 10.1002/aur.2696 PMC9310578

